# Iron-induced myocardial injury: an alarming side effect of superparamagnetic iron oxide nanoparticles

**DOI:** 10.1111/jcmm.12582

**Published:** 2015-06-04

**Authors:** Yunli Shen, Zheyong Huang, Xuebo Liu, Juying Qian, Jianfeng Xu, Xiangdong Yang, Aijun Sun, Junbo Ge

**Affiliations:** a Department of Cardiology, Shanghai East Hospital, Tongji UniversityShanghai, China; b Shanghai Institute of Cardiovascular Diseases, Zhongshan Hospital, Fudan UniversityShanghai, China

## Introduction and background

Superparamagnetic iron oxide nanoparticles (SPION), as magnetic resonance (MR) imaging contrast agents or magnetic targeting carriers, have potential applications in diagnostics, imaging, cell and drug/gene delivery for cardiovascular diseases. SPION are highly magnetic particles that cause magnetic field perturbations, which can be identified on T2* weighted images 1. Clinically, SPION allows non-invasive detection of the region of myocardial infarction and the peri-infarct zone based on a multiparametric cardiovascular MR approach 2,3, characterization of acute MI pathology by detecting infiltrating macrophages and altered perfusion kinetics 4 and non-invasive visualization of the aorta and aortic diseases 5. Preclinically, a large number of animal studies have been performed with SPION and cardiac magnetic resonance imaging to deliver, track or determine the efficacy of stem cell therapy in the heart in the past 10 years 1. More recently, magnetic targeting has emerged as a promising and novel strategy for ischaemic heart disease 6–10, in which SPION can direct drugs, genes or cells to the target site under a magnetic field gradient.

Superparamagnetic iron oxide nanoparticles’ biocompatibility with the target organ is the first prerequisite for clinical translation, and iron oxide nanoparticles have long been believed to have low toxicity and are well-tolerated in the human body. However, with the expanding application of SPION, toxic effects, such as oxidative stress and inflammatory reaction, have increasingly attracted attention. Iron oxide nanoparticles accumulate in lysosomes (following cellular internalization), in which the low pH breaks the iron oxide core down into iron ions. It has been reported that iron oxide nanoparticle inhalation exposure may induce lung cytotoxicity *via* oxidative stress and biphasic inflammatory responses in Wistar rats 11. *In vitro* studies have also suggested that iron oxide nanoparticles mediate activation of microglia in the brain 12 and differentiation of blood mononuclear cells into pro-inflammatory macrophages to secrete higher levels of pro-inflammatory cytokines 13. In addition, iron oxide particles stabilized with coatings, such as dextran or citric acid, induced toxic effects on the behaviour and function of endothelial cells 14–16 and activated the expression of genes related to oxidative stress 17. Moreover, the oxidative injury caused by SPION can be suppressed *via* antioxidant poly (trolox) nanoparticles binding to and internalizing in endothelial cells 16. Thus, could the invasion of SPION produce similar side effects in the myocardium?

Iron oxide nanoparticles with systemic administration were mainly cleared by the reticuloendothelial system and renal excretion, resulting in cytopathological effects on the lungs, liver and kidneys, while the heart and brain remain free from adverse effects because of limited iron deposits 18. A recent clinical study also showed that a single dose of intravenous iron oxide administration has a beneficial effect on LV remodelling in patients with acute ST-elevation myocardial infarction 19, in which the underlying iron deficiency with a decline in iron circulating levels was reported 20. However, this situation is quite different from local delivery of SPION-mediated therapeutic agents (stem cells, gene or drug) in the treatment of ischaemic heart disease. First, intramyocardial injection of SPION-mediated agents contains large amounts of iron oxide nanoparticles, and the local quantity of SPION deposition in the myocardium is higher than that reported in previous intravenous studies 21–23, in which SPION was administered systemically and proved to be a relatively safe and efficient MR contrast agent. Second, the heart is not a monocyte-macrophage organ, and iron clearance occurred more slowly in the heart than in the liver 24. Thus, it is difficult for macrophages to migrate away from the massive SPION introduced by SPION-mediated agents. Moreover, SPION-mediated therapeutic agents target the ischaemic or injured lesion rather than the normal myocardium. Thus, the injected SPION easily accumulates *in situ* for a prolonged period of time due to the lack of blood flow and mechanical contraction in the ischaemic or necrotic region 24,25. Magnetic resonance monitoring of SPION-containing stem cells in an animal model of myocardial infarction demonstrated that the iron particles could persist in the infarct lesion for several months 25,26. Third, this situation is even worse in the context of SPION-based magnetic targeting therapy introduced in cardiovascular diseases 6,7,27. Magnetic attraction could attenuate the loss of SPION-containing therapeutic drugs/cells *via* venous drainage, and subsequently increase the heart stay by approximately 3–10-fold 7. SPION may accumulate in the ischaemic myocardium in a highly clustered fashion when employed as magnetic carriers in targeting therapy. Thus, local delivery of SPION-mediated therapeutic agents might induce myocardial iron overload, particularly in the setting of myocardial infarction or magnetic targeting.

Another important question is whether SPION accumulation has toxicity effects on ischaemic myocardium. Although there is little information concerning the biological effects of SPION on myocardial tissues, the myocardium toxicity of excess non-SPION iron have been extensively explored. First, both primary haemochromatosis (a genetically determined condition resulting in iron overload) and secondary hemochromatosis (such as repeated transfusion, thalassaemia or sickle cell anaemia) can result in iron overload cardiomyopathy, with the pathogenic mechanism of that myocardial iron overload induces the formation of reactive oxygen species (ROS) *via* the Fenton reaction 28,29. The myocardium is one of the most sensitive tissues to iron, as demonstrated by the fact that myocardial injury and heart failure are a common presentation of hemochromatosis 24. In chronic iron overload, iron toxicity is dose-dependent 30. Second, recent studies have demonstrated that haemorrhagic myocardial infarction can result in local iron depositions within the infarct zones, which can be a source of prolonged inflammatory burden in the chronic phase of myocardial infarction, most likely resulting in LV negative remodelling 31 and ventricular arrhythmias 32. Third, acute myocardial ischaemia (specifically after reperfusion) can generate ROS *via* activation of the oxidative stress system 33 and then directly injuring the cell membrane of cardiomyocytes and induce cell death 34. SPION deposition might further enhance oxidative stress levels in ischaemic myocardium, thereby promoting more cardiomyocyte death.

The free radical-mediated pathway is the principal mechanism of iron toxicity in cardiomyocytes 35. Iron can be taken up by ventricular myocytes *via* the sarcolemmal L-type Ca^2+^ channel 36 in a dose- and time-dependent manner 37. Iron excess produces highly toxic hydroxyl radicals *via* the Fenton-catalysed Haber-Weiss reaction, which damages the lipid-rich cell membrane, and is known as lipid peroxidation. Cellular lipid peroxidation produces polyunsaturated fatty acids and increases toxic aldehydes. The aldehyde products can form a covalent link to proteins (aldehyde-protein adducts), rendering the loss of cell membrane integrity. Structures located on the cell membrane, such as Na^+^-K^+^ ATPase and 5′-nucleotidase, were affected thereafter. Oxidative stress-mediated iron toxicity also affects other cellular organelles and their functions. Consequently, iron-induced myocardial injury occurred.

## Hypothesis

Based on the available studies, it is logical to assume that local myocardial delivery of SPION-mediated therapeutic agents might produce myocardial iron overload, resulting in deterioration of myocardial injury and exacerbating cardiac function *via* oxidative stress-mediated iron toxicity, and undermining therapeutic effects. This hypothesis could be confirmed in an animal study. First, SPION-mediated therapeutic agents (such as SPION-labelled stem cells, *etc*.) are intramyocardially injected into peri-infarcted zones in an acute myocardial infarction rat model. Second, it should be investigated whether SPION deposition in the heart causes myocardiocyte loss and deteriorates the structure and function of the ventricle. For example, T2-star magnetic resonance (MR-T2*) was used to accurately evaluate cardiac iron status and detect early global ventricular dysfunction; lipid peroxidation products (8-iso-PGF2α and malondialdehyde, *etc*.) in the myocardium reflect the oxidative stress mechanism; and histology was performed to examine myocyte apoptosis, inflammation and fibrosis. Third, the efficacy of novel SPION coated with antioxidants (such as N-Acetylcysteine or Trolox) was investigated in attenuating oxidative stress-mediated cardiac injury, further validating the SPION’s adverse effects and its mechanism.

## Implication

The evaluation of SPION compatibility with myocardium, particularly with the ischaemic myocardium, is an urgent problem that needs to be resolved before the clinical translation of SPION in the cardiovascular field. If our hypothesis is true, then protective measures should be taken into consideration before developing clinical applications. Given that SPION toxicity mainly stems from oxidative stress, surface modification with an antioxidant (such as N-Acetylcysteine or Trolox) may be a new method used to suppress oxidative damage and injury.

In conclusion, local delivery of SPION-mediated therapeutic agents might produce massive and persistent iron overload in ischaemic myocardium, consequently deteriorating myocardial injury. Thus, antioxidant coating may be a new strategy used to suppress the harmful properties of SPION.
